# The Emerging Role of D-2-Hydroxyglutarate as an Oncometabolite in Hematolymphoid and Central Nervous System Neoplasms

**DOI:** 10.3389/fonc.2013.00169

**Published:** 2013-07-02

**Authors:** Dinesh Rakheja, L. Jeffrey Medeiros, Scott Bevan, Weina Chen

**Affiliations:** ^1^Department of Pathology, University of Texas Southwestern Medical Center and Children’s Medical Center, Dallas, TX, USA; ^2^Department of Pediatrics, University of Texas Southwestern Medical Center and Children’s Medical Center, Dallas, TX, USA; ^3^Department of Hematopathology, The University of Texas MD Anderson Cancer Center, Houston, TX, USA; ^4^AmeriPath/Quest Diagnostics, Dallas, TX, USA

**Keywords:** *IDH* mutation, *NPM1* mutation, acute myeloid leukemia, glioma

## Abstract

Approximately 20% of unselected cases and 30% cytogenetically diploid cases of acute myeloid leukemia (AML) and 80% of grade II–III gliomas and secondary glioblastomas carry mutations in the isocitrate dehydrogenase (IDH) 1 and 2 genes. *IDH1/2* mutations prevent oxidative decarboxylation of isocitrate to α-ketoglutarate (α-KG) and modulate the function of IDH (neomorphic activity) thereby facilitating reduction of α-KG to D-2-hydroxyglutarate (D-2HG), a putative oncometabolite. D-2HG is thought to act as a competitive inhibitor of α-KG-dependent dioxygenases that include prolyl hydroxylases and chromatin-modifying enzymes. The end result is a global increase of cellular DNA hypermethylation and alterations of the cellular epigenetic state, which has been proposed to play a role in the development of a variety of tumors. In this review, we provide an update on potential molecular mechanisms linking *IDH1/2* mutations and the resulting oncometabolite, D-2HG, with malignant transformation. In addition, in patients with AML and glioma we focus on the associations between *IDH1/2* mutations and clinical, morphologic, cytogenetic, and molecular characteristics.

## Introduction

There is increasing evidence that alterations in cellular metabolism are involved in the pathogenesis of many cancers. Notably, mutations in three different enzymes in the tricarboxylic acid cycle are associated with tumorigenesis. These enzymes are succinate dehydrogenease, fumarate hydratase, and isocitrate dehydrogenases (IDH1 and 2) (Soga, 2013). As many of the intermediates in the tricarboxylic acid cycle are important for synthesis of nucleotides, lipids, and amino acids, it is not surprising that alterations in this metabolic pathway may facilitate the development of cancers.

Somatic heterozygous mutations in *IDH1* and *IDH2* have been recognized recently in a number of cancers. The first reported mutation in an IDH-family gene was identified in a metastatic colon cancer in 2006 (Sjöblom et al., 2006). In 2008, sequencing of glioblastoma multiforme (GBM) tumor samples identified *IDH1* mutations at R132 (*IDH1*^R132^) in 12% of tumors (Parsons et al., 2008). In 2009, targeted mutational analysis of a broader group of central nervous system tumors detected *IDH1*^R132^ mutations in 70% of grade II and III gliomas (Yan et al., 2009). Also in 2009, whole-genome sequencing of a patient with acute myeloid leukemia (AML) identified an *IDH1*^R132^ mutation (Mardis et al., 2009). Subsequent analyzes on a large number of AML patients treated on multiple clinical protocols confirmed the presence of *IDH1*^R132^ mutations and also identified *IDH2*^R140^ and *IDH2*^R172^ mutations in ∼20% of adult-onset AML with normal cytogenetics (Mardis et al., 2009; Abbas et al., 2010; Boissel et al., 2010; Green et al., 2010; Gross et al., 2010; Ho et al., 2010; Marcucci et al., 2010; Paschka et al., 2010; Schnittger et al., 2010; Thol et al., 2010a). These mutations also have been identified, albeit at much lower frequency, in myelodysplastic syndromes and myeloproliferative neoplasms (Abbas et al., 2010; Green and Beer, 2010; Paschka et al., 2010; Tefferi et al., 2010; Thol et al., 2010b). Recently, a number of other tumors have been identified to harbor *IDH* mutations including angioimmunoblastic T-cell lymphoma (Cairns et al., 2012), chondrosarcoma (Amary et al., 2011), and intrahepatic cholangiocarcinoma (Borger et al., 2012; Wang et al., 2012).

*Isocitrate dehydrogenase 1* and *2* mutations reduce the affinity of their respective enzymes for isocitrate and increase their affinity for α-ketoglutarate (α-KG) and reduced nicotinamide adenine dinuceotide phosphate (NADPH). This reduced affinity impedes oxidative decarboxylation of isocitrate to α-KG and confers a novel enzymatic activity that facilitates reduction of α-KG to d-2-hydroxyglutarate (d-2HG) (Dang et al., 2009; Pietrak et al., 2011). Excess accumulation of d-2HG has been demonstrated in a subset of cases of glioma and AML with *IDH1* or *IDH2* mutations (Dang et al., 2009; Frezza et al., 2010; Gross et al., 2010; Ward et al., 2010; Andersson et al., 2011; Rakheja et al., 2011a; Choi et al., 2012). Others have suggested that overproduction of d-2HG promotes oncogenesis (Koivunen et al., 2012; Losman et al., 2013) and therefore *IDH1* and *IDH2* mutations are likely the integrally involved in the pathogenesis of malignant transformation (i.e., driver mutations) rather than epiphenomena.

In this review, we summarize the frequency and role of *IDH1* and *IDH2* mutations in gliomas and myeloid neoplasms, the latter with an emphasis on AML, and the association of these mutations with clinical, morphologic, cytogenetic, and molecular characteristics. We also provide an update on potential molecular mechanisms linking mutant *IDH1* and *IDH2* and their oncometabolite, d-2HG, with malignant transformation. The data suggest that *IDH1/2* mutations constitute an early mutational event which affects the cellular epigenetic state in a subset of gliomas and AML, an important consideration for the development of therapeutic agents.

### Biochemistry of isocitrate dehydrogenases

In mammalian cells, three classes of IDH isoenzymes exist: IDH1, IDH2, and IDH3 (Plaut et al., 1983). The human IDH1 enzyme is localized in the cytoplasm and peroxisomes and is encoded by *IDH1* at chromosome band 2q33.3 (Narahara et al., 1985; Geisbrecht and Gould, 1999). The IDH2 enzyme is localized in mitochondria and is encoded by *IDH2* at chromosome band 15q26.1 (Oh et al., 1996). The IDH1 and IDH2 enzymes are homodimeric, nicotinamide adenine dinucleotide phosphate-dependent, and catalyze the oxidative decarboxylation of isocitrate to α-KG, using NADP as a cofactor to yield NADPH (Haselbeck and McAlister-Henn, 1993). Mitochondrial NADPH participates in energy metabolism, as a part of the tricarboxylic acid cycle, and cytosolic NADPH, which is also produced in the pentose phosphate pathway, functions in anabolic processes and redox control. It is therefore reasonable to expect that changes in one or more of these processes occur in tumors that carry an *IDH1* or *IDH2* mutation. The NAD-dependent IDH3 is a heterotetramer composed of two alpha subunits (gene on chromosome 15), one beta subunit (gene on chromosome 20), and one gamma subunit (gene on chromosome X). IDH3 has not been shown to be mutated in cancer and therefore is not further addressed in this review.

### Neomorphic activity of IDH1/IDH2 mutant enzymes

Most cancer-associated enzyme mutations result in either catalytic inactivation or constitutive activation. A common feature of *IDH1* and *IDH2* mutations observed in AML and gliomas, however, is the apparent acquisition of enzymatic activity not shared by wild-type enzyme, known as neomorphic activity. The product of *IDH1*/*IDH2* mutations, d-2HG, can be detected in tumor samples.

To date, all reported *IDH1* or *IDH2* mutations are heterozygous, with cancer cells retaining one wild-type copy of the respective *IDH1* or *IDH2* allele. All *IDH* mutations identified involve a single amino acid substitution at an arginine (R) residue, R132 in *IDH1*, or R140 or R172 in *IDH2*; no inactivating (frame-shift or protein-truncation) mutations have been found. These residues create hydrophilic interactions that allow the binding of isocitrate (Xu et al., 2004). The residues that are substituted for arginine are wide ranging, and include histidine, serine, cysteine, glycine, or leucine, suggesting that it is the replacement of arginine, and not the specific amino acid substituted, that supports tumorigenesis.

**Figure 1:**
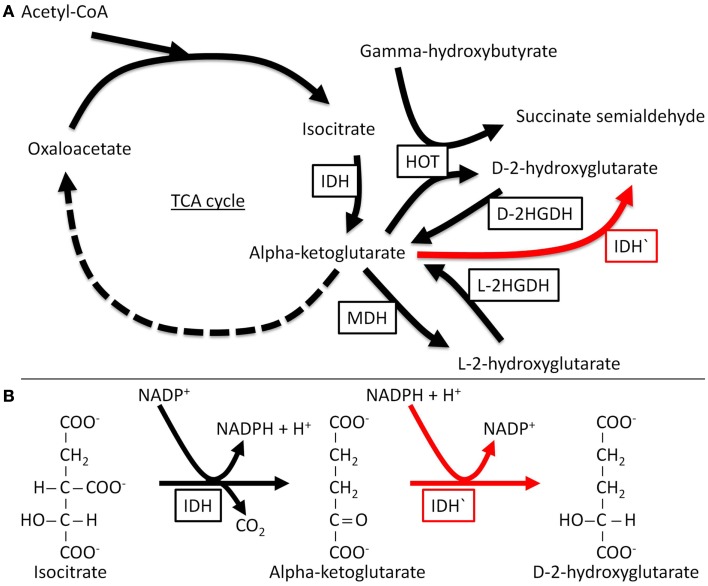
**Normal and neomorphic reactions catalyzed by IDH**. The normal enzyme catalyzes the nicotinamide adenine dinucleotide phosphate (NADP+)-dependent conversion of isocitrate to α-KG, while the mutant enzyme (neomorphic reaction in red) catalyzes the reduction of α-KG to D-2HG, a reaction that depends on NADPH (the reduced form of NADP+). D-2HGDH, D-2-hydroxyglutarate dehydrogenase; HOT, hydroxyacid oxoacid transhydrogenase; IDH, isocitrate dehydrogenase; IDH′, mutant isocitrate dehydrogenase; L-2HGDH, L-2-hydroxyglutarate dehydrogenase; MDH, malate dehydrogenase; TCA, tricarboxylic acid.

*In vitro* enzymatic analysis has confirmed that mutant IDH1 and IDH2 have altered substrate specificity and directionality (Dang et al., 2009; Zhao et al., 2009; Gross et al., 2010; Ward et al., 2010; Andersson et al., 2011; Pietrak et al., 2011). *IDH1* R132 and *IDH2* R172 are analogous residues that interact with the β-carboxyl of isocitrate. Normally, IDH functions as a homodimer; by contrast mutant IDH molecules in tumor cells form heterodimers with wild-type molecules. Whereas WT IDH converts isocitrate to α-KG, mutants of IDH can no longer catalyze this reaction and instead reduce α-KG to the d-stereoisomer of 2-hydroxyglutarate (d-2HG) (Figure 1). Structural comparisons of mutant and wild type IDH1 have revealed that mutations at R132 of *IDH1* result in impaired enzyme affinity for substrate and dominantly inhibit wild-type IDH1 activity through the formation of catalytically inactive heterodimers. The R132 mutation also changes the orientation of the catalytic site so that the enzyme binds NADPH and α-KG, explaining the formation of the new product, rather than simply catalyzing the reaction in reverse. In support of the results of *in vitro* enzymatic analyzes, d-2HG levels are 100-fold higher in cases of glioma and AML that carry *IDH1* or *IDH2* mutations as compared with tumors with wild type *IDH* (Dang et al., 2009; Gross et al., 2010; Ward et al., 2010; Andersson et al., 2011). Therefore, detecting d-2HG in tumor samples can reliably predict patients with tumor-associated *IDH* mutations (Gross et al., 2010).

### Mechanism of *IDH* mutations in tumorigenesis

The discovery of *IDH* mutations has led to renewed efforts to decipher the role of altered metabolic processes in cancer (Prensner and Chinnaiyan, 2011). The normal metabolic role of 2-hydroxyglutarate is not completely understood, but 2-hydroxyglutarate is not foreign to cells. It can be generated by specific α-KG reductase enzymes and oxidized back to α-KG by 2-hydroxyglutarate dehydrogenases (2HGDH). There are two enantiomers of 2-hydroxyglutarate with specific 2HGDH for each. Mutations in 2HGDH cause pathological accumulation of 2-hydroxyglutarate with different clinical features based on the enantiomer involved. Pathological accumulation of the l-2-hydroxyglutarate enantiomer (l-2HG) is known to occur in a rare inherited autosomal recessive disease characterized by encephalopathy and increased risk of brain tumors, including gliomas (Aghili et al., 2009). Accumulation of the d-2-hydroxyglutarate enantiomer (d-2HG) is associated with encephalopathy and cardiomyopathy, but not with tumors (Struys, 2006). Mutant IDH in cases of AML and glioma generates d-2HG and not the l-enantiomer.

As a result of *IDH* mutation, the oncometabolite of d-2HG is produced and α-KG is reduced. The combination of these two events may be important. At the high levels of d-2HG that have been observed in cases of AML and glioma (more than 10 mM) (Dang et al., 2009; Gross et al., 2010; Ward et al., 2010; Andersson et al., 2011), d-2HG may lead to DNA damage mediated by elevated levels of reactive oxygen species (Zhao et al., 2009; Ward et al., 2010), induction of redox stress owing to impairment of the respiratory chain, promotion of oncogenesis by promoting cytokine independence, and blocking differentiation in hematopoietic cells (Losman et al., 2013). Notably, d-2HG and α-KG are structurally similar except that the oxygen atom linked to C2 in α-KG is replaced by a hydroxyl group in d-2HG. This structural similarity suggests that d-2HG might exert its oncogenic effects through competitive inhibition with α-KG-dependant dioxygenases (Figueroa et al., 2010; Chowdhury et al., 2011; Xu et al., 2011). These enzymes include prolyl hydroxylases, and chromatin-modifying enzymes, such as histone demethylases and TET 5-methylcytosine hydroxylases.

TET2 is an α-KG-utilizing enzyme that hydroxylates 5-methylcytosine as a step in demethylation of DNA (Ito et al., 2010). Recently, all TET family members including TET2 were shown to catalyze the conversion of 5-methylcytosine to 5-hydroxymethylcytosine (5-OH-MeC) (Ito et al., 2010). The fact that *IDH1* and *IDH2* mutations are mutually exclusive, and that *IDH1/2* mutations are mutually exclusive with *TET2* mutations, suggests that the biological effects of mutant IDH1/2 and TET are similar (Figueroa et al., 2010; Chou et al., 2011a; Patel et al., 2012). AMLs with *TET2* or *IDH* mutation, and *IDH* mutated gliomas have more pronounced hypermethylation profiles than their non-mutated counterparts and share overlapping epigenetic signatures (Figueroa et al., 2010; Xu et al., 2011). Most importantly, expression of IDH1/2 mutants induces a global increase in global DNA hypermethylation and inhibits TET2-induced cytosine 5-hydroxymethylation due to reduction of α-KG. These data suggest that *TET2* and *IDH1/2* mutations characterize a distinctive group of AML cases in which the epigenetic state is altered. Many of the genes hypermethylated in the context of *IDH1/2* mutated AML contain DNA-binding motifs for GATA1/GATA2 and EVI1, transcription factors known to play a role in leukemogenesis as well as normal myeloid differentiation (Figueroa et al., 2010).

Recent analysis of gliomas also has shown that *IDH1* gene mutations represent a molecular basis for the epigenetic changes described above. In one study, the methylation profiles of immortalized primary human astrocytes in which either wild-type *IDH1* or R132H *IDH1* was introduced were analyzed using a genome-wide platform. Expression of wild-type IDH1 led to hypomethylation at several loci, compared with a marked increase in methylation seen in the R132H *IDH1* mutants. The methylation profiles of the *IDH1* mutants mirrored the CpG island methylator phenotype (CIMP), a unique phenotype seen in several tumors with extensive epigenetic changes (Turcan et al., 2012).

Histone lysine demethylases are another group of α-KG-dependent enzymes. *In vitro*, d-2HG can inhibit histone demethylases (Chowdhury et al., 2011; Xu et al., 2011). Experimental data suggest that inhibition of histone demethylases can induce DNA methylation (Lu et al., 2012). Methylated histones, such as H3K9me3 (trimethylation of lysine 9 of histone H3), may serve as a platform for recruiting a complex of heterochromatin protein 1 (HP1), histone methyltransferase, and DNA methytransferase. Direct cooperation between these enzymes could then provide a mechanism of coordinated histone and DNA methylation involved in epigenetic regulation of DNA (Estève et al., 2006).

Hypoxia-inducible factor 1α (HIF-1α) is a transcription factor that has functions linked to metabolism, angiogenesis, and tumorigenesis. HIF-1α protein levels are downregulated under normoxic conditions by prolyl hydroxylase-mediated hydroxylation and subsequent hydroxylation-targeted ubiquitination (Bruick and McKnight, 2001). As α-KG is required by prolyl hydroxylases, a reduction in α-KG levels in cancer cells with mutant IDH may lead to inhibition of prolyl hydroxylases and stabilization of HIF-1α (Zhao et al., 2009). An alternative mechanism proposed by Koivunen et al. (2012) suggests that d-2HG acts as a cofactor to promote the hydroxylase activity of the Eg1N prolyl-4-hydroxylase and subsequent downregulation of HIF1α, contributing to the pathogenesis of IDH mutant gliomas (Koivunen et al., 2012). These data seem incompatible with the finding that HIF1α protein levels are increased in IDH mutant tumors. However, these data may suggest that d-2HG quantitatively shifts the dose-response linking HIF activation to hypoxia, leading to a blunted HIF response for a given level of hypoxia. In support of this idea, HIF elevation in IDH mutant tumors is usually confined to areas of necrosis and presumed severe hypoxia (Williams et al., 2011).

Collectively, the data support the concept that *IHD* mutations promote oncogenesis through d-2HG-induced inhibition of α-KG-dependent enzymes, such as TET2 and histone demethylase. This idea further suggests a paradigm whereby oncogenic alterations in core cellular metabolic pathways could lead to neoplastic progression by dysregulating the epigenetic machinery in hematopoietic and glial progenitors. Mutations of *IDH1* and *IDH2* in combination with microenvironmental effects in certain tumor types are likely the driver mutations that are responsible for the malignant phenotype, rather than simply epiphenomena (Dang et al., 2009; Figueroa et al., 2010; Xu et al., 2011; Koivunen et al., 2012; Losman et al., 2013).

### *IDH* mutations in AML

Mardis and colleagues (Mardis et al., 2009) used next generation (massive parallel) DNA sequencing analysis of the genome of an AML patient with a normal karyotype and discovered an *IDH1* mutation. Subsequently, this finding was validated in 187 AML patients in whom 8% had *IDH1* mutations. To date, there have been ∼20 published studies on *IDH1* and *IDH2* mutations that have included ∼8,000 adult and ∼800 pediatric patients (Mardis et al., 2009; Abbas et al., 2010; Boissel et al., 2010; Chou et al., 2010, 2011b; Figueroa et al., 2010; Gross et al., 2010; Marcucci et al., 2010; Paschka et al., 2010; Schnittger et al., 2010; Thol et al., 2010a; Wagner et al., 2010; Ward et al., 2010; Andersson et al., 2011; Damm et al., 2011; Green et al., 2011; Pigazzi et al., 2011; Rakheja et al., 2011a; Patel et al., 2012). These studies have focused on the frequency and prognostic influence of *IDH* mutations in the context of other genetic mutations and prognostic markers.

*Isocitrate dehydrogenase 1/2* mutations are almost mutually exclusive in AML, as only rare cases (<0.5%) harbor both *IDH1* and *IDH2* mutations (Abbas et al., 2010; Paschka et al., 2010; Green et al., 2011). This low frequency of concurrent mutations suggests that the biological effects of *IDH1* and *IDH2* are similar, and this idea is corroborated by the similar impact of these mutations on the distribution of cytosine methylation and the global DNA methylation profile in AML cells (Figueroa et al., 2010).

For *IDH1*^R132^ mutations in AML, as in other tumors, five major different amino acid substitutions for arginine (R) have been detected: cysteine (R132C), leucine (R132L), glycine (R132G), histidine (R132H), and serine (R132S) (Mardis et al., 2009; Abbas et al., 2010; Chou et al., 2010; Ho et al., 2010; Marcucci et al., 2010; Schnittger et al., 2010; Wagner et al., 2010; Patel et al., 2011a). R132C (∼30%) and R132H (∼50%) are the most common mutations in AML. The mutational profile is slightly different in gliomas, in which R132H (∼90%) is most common and R132C is uncommon (∼4%) (Yan et al., 2009; Chou et al., 2010). For *IDH2*^R140^ mutations, three different amino acid substitutions have been detected: glutamine (R140Q), leucine (R140L), and tryptophan (R140W). Of these mutations, R140Q is the most common by far, seen in ∼95% of AMLs with mutated *IDH2* at R140. For *IDH2*^R172^ mutations, almost all involve replacement of arginine by lysine (R172K), except rare cases in which arginine is replaced by methionine (R172M) (Chou et al., 2011b).

In unselected adults with AML, in a total of 6,877 patients, *IDH1* and *IDH2* mutations have been identified in 7.3 and 9.7% of cases, respectively. The frequencies of *IDH1* and *IDH2* mutations are higher in cytogenetically normal (CN) versus abnormal AML patients (11 versus 3.5% for *IDH1* mutations; and 16 versus 3.8% for *IDH2* mutations), placing them among the most common molecular aberrations in CN-AML. Within the group of *IDH2* mutations, *IDH2*^R140^ mutations are most common (∼80%) whereas *IDH2*^R172^ mutations occur in ∼2% of unselected AML and about 2.5% of CN-AML patients.

Acute myeloid leukemia patients with *IDH1*^R132^ or *IDH2*^R140^ mutations are more frequently older at diagnosis as compared with wild type AML patients. AML patients with *IDH2*^R172^ mutation are usually older with lower white blood cell (WBC) counts. The frequency of *IDH1/2* mutations is substantially lower in pediatric (∼1–2%) than in adult patients with AML. *IDH2*^R172^ mutations have not been observed in children (Marcucci et al., 2010). In most studies to date, there has been little focus correlating *IDH1/2* mutations with morphologic findings in AML. Most cases with *IDH1/2* mutations have been classified as AML, not otherwise specified, with or without maturation. A rare morphologic subset of AML characterized by cuplike nuclei appears to commonly carry *IDH1/2* mutations. In one study, 8 of 12 (67%) patients with AML with cuplike nuclei patients harbored either *IDH1^*R132*^* or *IDH2^*R140*^* mutations (Rakheja et al., 2011a). AML with cuplike nuclei also have a high frequency of *NPM1* mutations and *FLT3* internal tandem duplication (*FLT3*-ITD) (Chen et al., 2009).

Patients with AML carrying *IDH1/2* mutations have a higher frequency of *NPM1* mutations compared with AML wild type for *IDH1/2*. In general, *IDH1/2* mutations are less frequent in AML patients with activating *FLT3* mutations, *CEBPA* mutations, and are largely absent in patients with AML associated with recurrent chromosomal abnormalities, such as *t*(15;17), *t*(8;21), inv(16) (Chou et al., 2010, 2011b; Patel et al., 2011a). *IDH2*^R172^ mutation also appears to be virtually mutually exclusive with other genetic abnormalities (Marcucci et al., 2010; Paschka et al., 2010; Green et al., 2011; Patel et al., 2011a), thereby identifying a novel subset of patients among the ∼3% of CN-AML adult patients for whom no prognostic gene mutation has been reported to date.

The prognostic effect of *IDH* mutations in adult AML patients has been intensively studied, but remains a matter of discussion. In general, there have been no differences in response to therapy and survival between *IDH1/2*-mutated versus *IDH1/2*-wild type AML patients (Abbas et al., 2010; Chou et al., 2010; Thol et al., 2010a; Wagner et al., 2010; Marcucci et al., 2011). The impact of an *IDH1* or *IDH2* mutation, however, does seem to have prognostic importance if results are stratified according to cytogenetic data, *FLT3*-ITD and *NPM1* mutation status, and type of *IDH* mutation (Boissel et al., 2010; Green et al., 2010, 2011; Marcucci et al., 2010; Paschka et al., 2010; Schnittger et al., 2010). *IDH1* mutations may predict higher risk of relapse and shorter survival in the subset of CN-AML patients with mutated *NPM1* and absence of *FLT3*-ITD (molecular low-risk group) (Marcucci et al., 2010; Paschka et al., 2010). In addition, there is some evidence that *IDH2*^R172^ mutations confer a poorer prognosis whereas *IDH2*^R140^ mutations have no impact on survival or may confer a favorable outcome (Boissel et al., 2010; Marcucci et al., 2010; Chou et al., 2011b; Green et al., 2011; Patel et al., 2012). Future prospective studies and clinical trials are needed to assess the prognostic impact of each type of *IDH* mutation in AML patients within the context of other molecular aberrations.

In pediatric AML patients, there is an association between *NPM1* and *IDH* mutations, as has been described in adult AML patients. However, in contrast with adults, *IDH* mutations are not associated with a normal karyotype in childhood AML. Instead, *IDH* mutations have been observed in pediatric AML patients with good-risk cytogenetics, *t*(8;21), and *t*(15;17). In addition, neither type of *IDH* mutation occurs in children<3 years, affirming the distinctiveness of infant AML.

### *IDH* mutations in myelodysplastic syndromes and myeloproliferative neoplasms

*Isocitrate dehydrogenase* mutations occur at low frequency in patients with myelodysplastic syndromes (3.6–5%) and patients with Philadelphia chromosome/*BCR-ABL1* negative myeloproliferative neoplasms in chronic phase (2–4%). Mutated cases have an increased frequency of progression to AML, ranging from 7.5 to 31% in different studies (Abbas et al., 2010; Green and Beer, 2010; Pardanani et al., 2010; Tefferi et al., 2010, 2011; Thol et al., 2010b). In patients with myeloproliferative neoplasms characterized by *JAK2* and *IDH1/2* mutations, it is tempting to speculate that these gene mutations have an additive or cooperative effect to facilitate leukemogenesis. The *JAK2* mutation may offer a proliferative advantage whereas *IDH1/2* mutations may disrupt epigenetic remodeling. Mutations in *IDH1* or *IDH2* mutations are absent or very rare in patients with chronic myelogenous leukemia in chronic phase (Abbas et al., 2010).

### *IDH* mutations in gliomas

The first report of an *IDH* mutation in gliomas occurred in 2008 after over 20,000 protein coding genes were analyzed in 22 human GBM samples (Parsons et al., 2008). In this study, *IDH1*^R132^ point mutations were detected in 12% of GBM samples. The authors observed that *IDH1* mutations occurred in younger adults, in most of the secondary GBM samples, and that mutation was associated with increased overall survival. The fact that *IDH1* mutations occurred in secondary GBM prompted the same group to determine the frequency of *IDH1* mutations in the low-grade gliomas. Targeted sequencing of *IDH1* and *IDH2* in 445 central nervous system tumors revealed *IDH1/2* mutations in 90% of cases of diffuse astrocytoma (grade II), 84% of oligodendroglioma (grade II), 73% of anaplastic astrocytoma (grade III), 94% of anaplastic oligodendroglioma (WHO grade III), and 85% of secondary GBM (grade IV) (Yan et al., 2009). Several follow-up studies encompassing thousands of central nervous system neoplasms have found similar frequencies of *IDH1* and *IDH2* mutations (Hartmann et al., 2009; Nobusawa et al., 2009; van den Bent et al., 2010; Jha et al., 2011; Thon et al., 2012). Similar to what has been observed in AML patients, *IDH1* and *IDH2* gene mutations in gliomas are mutually exclusive, heterozygous, and restricted to the R132 site of *IDH1* and the R172 site of *IDH2*. The discovery of *IDH* mutations in gliomas has allowed for further characterization of the sequence of events in glioma pathogenesis. The detection of *IDH* mutations in most low-grade gliomas and secondary GBM suggests that *IDH1* mutation is an early event in pathogenesis. Cases of primary GBM that do not show *IDH* mutations are genetically distinct.

### Potential utility of detecting *IDH* mutations in gliomas

Detection of an *IDH* mutation, either directly in surgical specimens or indirectly by measuring d-2HG levels in the brain, has practical implications. The presence of *IDH* mutation in a GBM in a patient without a prior history of a brain lesion would support a diagnosis of secondary GBM, which portends a better, albeit still dismal prognosis. Like primary GBM, grade I central nervous system tumors, such as pilocytic astrocytoma, and non-neoplastic brain tissue lack *IDH* mutations. Distinguishing rare infiltrating neoplastic cells from reactive gliosis in surgical biopsy specimens can be difficult, and the presence of *IDH* mutation would support a neoplastic process. An additional implication includes distinguishing diffuse astrocytoma (grade II) from pilocytic astrocytoma (grade I). Although associated with a better prognosis, the presence of *IDH* mutations in gliomas does not predict a response to therapy (Capper et al., 2010; Preusser et al., 2011).

### Potential utility of *IDH* mutations as a follow-up marker

*Isocitrate dehydrogenase 1* and *2* mutations appear to be stable. In one study of 225 AML patients with wild type *IDH* at diagnosis, not a single patient acquired an *IDH* mutation during clinical follow-up (Chou et al., 2010). In addition, in patients with *IDH* mutated AML at diagnosis, the same mutation persisted in over 95% of *IDH1*-mutated and *IDH2*-mutated AML cases at relapse (Chou et al., 2010, 2011b; Schnittger et al., 2010; Thol et al., 2010a). From a biological perspective, this stability suggests that *IDH* mutations may be a primary event that is involved early in leukemogenesis and/or maintenance of the leukemic phenotype. Recent data (Dinardo et al., 2013) suggest that elevated levels of d-2HG in serum, higher than 700 ng/ml could segregate patients with and without *IDH* mutations. Furthermore, *IDH* mutant patients with d-2HG levels>200 ng/ml at complete remission experienced shorter overall survival compared to those with d-2HG<200 ng/ml. These data confirm that serum measurement of an oncometabolite can provide useful information for diagnosis, treatment response, and prognosis.

### Methods of detecting *IDH* mutations and the oncometabolite d-2HG

*Isocitrate dehydrogenase* mutations can be detected by a number of methods including polymerase chain reaction (PCR)-based assays (Felsberg et al., 2010; Meyer et al., 2010; Patel et al., 2011b; Rakheja et al., 2011a), expression of IDH1 mutant protein in tumor tissue as detected by mutation specific antibody using immunohistochemistry (Andrulis et al., 2010; Capper et al., 2010), and indirectly by detecting the putative oncometabolite, d-2HG, by mass-spectrometry *in vitro* or by magnetic resonance spectroscopy *in vivo* (Gross et al., 2010; Rakheja et al., 2011b; Choi et al., 2012; Sahm et al., 2012).

Polymerase chain reaction-based assays include amplification of genomic DNA at exon 4 of *IDH1* (*IDH1*^R132^) and *IDH2* (*IDH2*^R140^ or *IDH2*^R172^) followed by Sanger sequencing, restriction endonuclease digestion and high-resolution melting curve analysis, and pyrosequencing. Unfortunately, these methods have a relatively low sensitivity, for example, ∼20% for heterozygous mutations by Sanger sequencing. More sensitive methods, such as allele-specific PCR, also can be performed to detect of *IDH1/IDH2* mutations. More recently, next generation sequencing methods have been applied to the detection of *IDH1/2* mutations. With coverage of 500 times, the sensitivity of this approach is ∼5%. These more sensitive assays can be used to detect mutations in treated AML patients in whom bone marrow samples have low blast counts as well as for the detection of early relapse or minimal residual disease.

Currently, only IDH1 mutant protein, IDH1^R132^ can be detected by immunohistochemistry using the H09 clone (Dianova, Hamburg, Germany) (Andrulis et al., 2010; Capper et al., 2010; Sahm et al., 2012). This antibody has been examined in gliomas, but has not yet been evaluated comprehensively in the myeloid neoplasms. Future development of a monoclonal antibody targeted at the IDH2 mutant proteins, IDH2^R140^ or IDH2^R172^, would be helpful and convenient for detecting these mutations.

In the laboratory at Children’s Medical Center of Dallas, exon 4 and flanking intronic regions of *IDH1* and *IDH2* are sequenced (Rakheja et al., 2011a,b,c). PCR amplification is followed by Sanger sequencing. At MD Anderson Cancer Center, a Sanger based assay has been recently transitioned to a next generation sequencing assay used to detect *IDH1/2* mutations in AML patients at time of initial diagnosis. The metabolites, d-2HG and l-2-hydroxyglutaric acid (l-2HG), are measured using liquid chromatography-tandem mass spectrometry (LC-MS/MS) as described previously (Rakheja et al., 2011a,b,c).

Detection of d-2HG in tumor tissue by mass-spectrometry *in vitro* or by magnetic resonance spectroscopy *in vivo* offers an advantage in that potentially all mutations are detectable, as all mutations generate this oncometabolite (Gross et al., 2010; Rakheja et al., 2011b,c; Choi et al., 2012; Sahm et al., 2012). This non-invasive detection of d-2HG may prove to be valuable in diagnosis and providing prognostic biomarker. It should be noted that archival formalin-fixed paraffin-embedded tumor specimens may not be optimal for detecting d-2HG because this metabolite can be lost during the routine embedding process (Sahm et al., 2012).

### Implications for future treatment

*Isocitrate dehydrogenase 1*/*2* mutations are thought to be one of the driver mutations in AML and early events in the pathogenesis of gliomas. The common feature of *IDH1* and *IDH2* mutations is the ability of the respective enzymes to exhibit neomorphic activity, not characteristic of the wild type enzymes, that facilitate production of d-2HG. Therefore, targeted therapies that inhibit the neomorphic function of mutant IDH enzymes or DNA hypomethylating agents might reverse the associated epigenetic patterning and may promote myeloid or glial differentiation and improve outcome in patients with *IDH1/2*-mutated tumors. Recently, *IDH* inhibitors have been shown to produce cytostatic effects and cellular differentiation in leukemia and glioma cells (Rohle et al., 2013; Wang et al., 2013). It is currently unknown, however, whether these inhibitors can induce a permanent state of differentiation. The survival of viable tumor cells still containing a potentially transforming constellation of mutations makes it important to determine whether the therapeutic effects will persist over long time frames.

### Conclusions and future perspectives

*IDH1*^R132^, *IDH2*^R140^, and *IDH2*^R172^ mutations represent a novel class of point mutations in patients with AML and glioma. In AML patients, it is possible that *IDH1*^R132^, *IDH2*^R140^, and *IDH2*^R172^ mutations represent molecular or clinically distinctive subgroups, with *IDH1*^R132^ and *IDH2*^R140^ more frequently accompanied by normal cytogenetics and *NPM1* mutation, whereas *IDH2*^R172^ is frequently the only mutation detected and portends a poor prognosis. In glioma patients, *IDH* mutations are present in grade II and III gliomas as well as secondary GBM. Whether these different mutation types represent distinctive subgroups of glioma patients is less understood. As both *IDH1* and *IDH2* mutations result in the generation of the putative oncometabolite, d-2HG, this oncometabolite can be measured directly in tumor samples and serum. Screening for the presence of d-2HG could be used as an assay to detect *IDH* mutations, monitor therapeutic response, and potentially uncover novel *IDH* mutations. Importantly, expression of mutant IDH1/2 induces a global increase in DNA hypermethylation and inhibits TET2-induced cytosine 5-hydroxymethylation, suggesting that *TET2* and *IDH1/2* mutations constitute a distinct mutational class in these tumors in which the epigenetic state is altered. Furthermore, this biologic effect is an important consideration for developing a therapeutic agent that can target dysregulated IDH enzymes in addition to induction of DNA hypomethylation. Inhibitors to mutant IDH have been shown recently to produce cytostatic effects and cellular differentiation in leukemia and glioma cells and seem promising. Future studies are important to determine whether these inhibitors can induce a permanent state of differentiation, and their therapeutic effects and toxicity in clinical trials. Discovering *IDH* mutations is an example of a pathologic finding that links disruption of metabolism to oncogenesis.

## Conflict of Interest Statement

The authors declare that the research was conducted in the absence of any commercial or financial relationships that could be construed as a potential conflict of interest.
